# Gaps in the usage and reporting of multiple imputation for incomplete data: findings from a scoping review of observational studies addressing causal questions

**DOI:** 10.1186/s12874-024-02302-6

**Published:** 2024-09-04

**Authors:** Rheanna M. Mainzer, Margarita Moreno-Betancur, Cattram D. Nguyen, Julie A. Simpson, John B. Carlin, Katherine J. Lee

**Affiliations:** 1 Clinical Epidemiology and Biostatistics Unit, Murdoch Children’s Research Institute, Parkville, Victoria 3052 Australia; 2 Department of Paediatrics, The University of Melbourne, Parkville, Victoria 3052 Australia; 3 Centre for Epidemiology and Biostatistics, Melbourne School of Population and Global Health, The University of Melbourne, Parkville, Victoria 3052 Australia; 4 Nuffield Department of Medicine, University of Oxford, Oxford, UK

**Keywords:** Missing data, Causal inference, Missingness mechanism

## Abstract

**Background:**

Missing data are common in observational studies and often occur in several of the variables required when estimating a causal effect, i.e. the exposure, outcome and/or variables used to control for confounding. Analyses involving multiple incomplete variables are not as straightforward as analyses with a single incomplete variable. For example, in the context of multivariable missingness, the standard missing data assumptions (“missing completely at random”, “missing at random” [MAR], “missing not at random”) are difficult to interpret and assess. It is not clear how the complexities that arise due to multivariable missingness are being addressed in practice. The aim of this study was to review how missing data are managed and reported in observational studies that use multiple imputation (MI) for causal effect estimation, with a particular focus on missing data summaries, missing data assumptions, primary and sensitivity analyses, and MI implementation.

**Methods:**

We searched five top general epidemiology journals for observational studies that aimed to answer a causal research question and used MI, published between January 2019 and December 2021. Article screening and data extraction were performed systematically.

**Results:**

Of the 130 studies included in this review, 108 (83%) derived an analysis sample by excluding individuals with missing data in specific variables (e.g., outcome) and 114 (88%) had multivariable missingness within the analysis sample. Forty-four (34%) studies provided a statement about missing data assumptions, 35 of which stated the MAR assumption, but only 11/44 (25%) studies provided a justification for these assumptions. The number of imputations, MI method and MI software were generally well-reported (71%, 75% and 88% of studies, respectively), while aspects of the imputation model specification were not clear for more than half of the studies. A secondary analysis that used a different approach to handle the missing data was conducted in 69/130 (53%) studies. Of these 69 studies, 68 (99%) lacked a clear justification for the secondary analysis.

**Conclusion:**

Effort is needed to clarify the rationale for and improve the reporting of MI for estimation of causal effects from observational data. We encourage greater transparency in making and reporting analytical decisions related to missing data.

**Supplementary Information:**

The online version contains supplementary material available at 10.1186/s12874-024-02302-6.

## Background

Observational studies in medical and health-related research often aim to answer a causal question, which we understand as estimation of the average causal effect (ACE) of an exposure on an outcome in a population of interest [1, 2]. Missing data in observational studies often occurs in multiple variables required for the estimation of ACEs, such as the exposure, the outcome and/or the covariates used to control for confounding. Applying standard methods for ACE estimation (e.g., outcome regression with covariate adjustment) using only data from complete records (“complete cases analysis” [CCA]) may lead to selection bias and also overlook precision gains that might be achieved by incorporating information from the incomplete cases [3] . Therefore, missing data need to be carefully considered and addressed to minimise the potential for selection bias and loss of information.

One flexible and widely recommended approach for estimation in the presence of multivariable missingness is multiple imputation (MI) [4–6]. In the first stage of MI, missing data are imputed multiple times with random draws from the predictive distribution of the missing values given the observed data and a specified imputation model. In the second stage, the statistical analysis of interest (e.g., outcome regression with covariate adjustment) is applied to each imputed dataset and the results are combined to obtain a single estimate with associated standard error [4].

To date most reviews of the handling of missing data or the application of MI have been carried out in the context of trials (see [7] and references therein). In contrast, there has been little attention given to how missing data are handled in observational studies, a context in which multivariable missingness is often encountered. Mackinnon (2010) and Hayati Rezvan et al. (2015) reviewed the implementation and documentation of MI in both trials and observational studies [8, 9], and Karahalios et al. (2012) reviewed how missing exposure data are reported in large cohort studies with one or more waves of follow-up [10]. More recently, Carroll et al. (2020) reviewed the handling of missing covariates in observational time-to-event studies in oncology [11], Okpara et al. (2022) reviewed the handling of missing data in longitudinal studies of older adults [12], and Benneville et al. (2023) reviewed the handling of missing covariate data in the field of haematology [13]. However, none of the above reviews focussed on the complexities that arise due to multivariable missingness in exposure, outcome and covariates.The aim of the current study was to review the handling of missing data in observational studies that address causal questions using MI. A scoping review was conducted to systematically benchmark the current state of practice [14], focussing on four key areas: missing data summaries, missing data assumptions, primary and sensitivity analyses, and MI implementation. In the next section we describe considerations for transparent reporting within each of these four areas to provide context for our review. We then describe our scoping review methodology and present our results. We end with a discussion of our findings and key messages.

### Considerations for reporting ACE estimation with MI from incomplete observational data

Several frameworks and guidelines around missing data and the application of MI are available (see [15, 16] and Table 1 of [11]. In this section we outline key considerations when estimating and reporting ACEs from incompletely observed data that are pertinent to the current review from these guidelines.

### Missing data summaries

Describing the amount of missing data is an important first step for transparent reporting as the potential for selection bias will generally increase with larger proportions of missing data. When data are missing in a single variable, the number (%) of completely observed values for that variable also summarises the number (%) of complete cases. In contrast, when multiple variables required for analysis are incompletely observed, the number (%) of observed values for each variable may vastly differ from the number (%) with complete cases because of the pattern of missing data, that is, the way in which the variables are jointly missing. In the latter context, a complete description of the missing data would include summaries of the missing data for each variable, as well as summaries of the distinct missing data patterns. Such summaries can be easily obtained in statistical software.

### Missing data assumptions

Understanding the process that cause data to be missing, i.e., the “missing data mechanism”, is important because the performance of any estimation method depends critically on this. Sometimes the missing data mechanism will be known (e.g. a machine used for measurement temporarily stopped working), but in most cases it will be unknown and assumptions about the mechanism, along with a justification for these assumptions, are required. Missingness assumptions are often expressed using the classification of missing data patterns as “missing completely at random” (MCAR), “missing at random” (MAR) or “missing not at random” (MNAR) [16, 17]. However, assessing the plausibility of the MCAR/MAR/MNAR assumptions in the context of multivariable missingness is difficult, partly due to the existence of several different, often imprecise, definitions of MCAR, MAR and MNAR in the literature and the difficulty of interpreting these definitions, [18] and partly because assessment involves making a judgement about the dependence (or lack thereof) of the distribution of the missing data pattern on the observed and missing data [16, 19]. An attractive alternative to using the MCAR/MAR/MNAR framework is to view missing data as a causal problem and to represent assumptions about causes of missingness for each incompletely observed variable using missingness directed acyclic graphs (m-DAGs) [3, 20]. m-DAGs are an extension to standard causal diagrams (DAGs) that include nodes to represent missingness in each incomplete variable, thereby allowing for the clear and transparent specification of assumptions about the causes of missing data, as well as the causal relationships amongst the main variables of interest. Assumptions about the causes of missingness can be justified using expert knowledge, literature or external data (see, e.g., Fig. 3 of [3] and Table 5 of [21]). Although developing a realistic m-DAG can be challenging and time-consuming, m-DAGs lead to assumptions that are more transparent and easier to assess than assumptions expressed using the MCAR/MAR/MNAR framework. Uncertainty about the assumptions depicted in the m-DAG can be assessed using a sensitivity analysis (see next section).

### Primary and sensitivity analyses

The next important area for reporting is to justify and describe an appropriate primary method for estimation of the ACE, given the missingness assumptions. It is well known that both a CCA and standard MI (an implementation of MI that does not incorporate an external assumption about a difference between the distribution of the observed and missing data) can provide consistent estimation of the ACE when data are MCAR, that standard MI can provide consistent estimation when data are MAR, and that both approaches may provide biased estimation when data are MNAR. However, in the context of multivariable missingness, a CCA can also provide consistent estimation under missingness mechanisms that could be classified as MAR, and both CCA and MI have been shown in theory and simulations to provide unbiased or approximately unbiased estimation of ACEs across a range of missingness mechanisms that could be classified as MNAR [21]. Therefore, it is not straightforward to justify an estimation approach even if it is believed that data are MAR or MNAR. In contrast, for a given m-DAG, graph theory can be used to establish whether the ACE is *recoverable* (that is, whether it can be estimated unbiasedly from the observed data). If the ACE is recoverable, the process of establishing recoverability can aid in determining whether a CCA and/or standard MI would be appropriate for estimation (see, e.g., the worked example provided by Lee et. al. [16]). If the ACE is not recoverable, neither standard MI nor a CCA can be used for unbiased estimation, and a more sophisticated approach that incorporates an assumption about a difference in distribution between the missing and observed values is needed. For example, the not-at-random fully conditional specification (NARFCS) procedure extends standard MI to incorporate such assumptions through the inclusion of a sensitivity parameter “delta”, elicited from external information, that represents the difference between the distributions of the observed and missing values [22]. The assumptions made about the missing data and how this justifies the choice of analytic method for the primary analysis should be carefully described.

Sensitivity analyses to reflect uncertainty due to assumptions made about the missing data for the primary analysis are strongly recommended [15, 23]. There are two types of missing data sensitivity analyses to consider; the first is to examine the sensitivity of estimates to the assumptions made about the causes of missing data, e.g. the existence or strength of arrows in the m-DAG. The second type of missing data sensitivity analysis is to examine the sensitivity of estimates to assumptions made for modelling the missing data, such as the form of the imputation model (e.g., linear regression vs predictive mean matching for imputing continuous variables). As with the primary analysis, sensitivity analyses should be justified and described in enough detail that the analysis could be reproduced.

### MI implementation

When using standard MI for estimation, quantities that need to be described to ensure that the analysis could be reproduced include, but are not limited to: the imputation method, e.g., multivariate normal imputation or multivariate imputation by chained equations; the imputation model, e.g., which variables are included and in what form; if using multivariate imputation by chained equations, the models/methods that are used to impute each incomplete variable, e.g., linear or logistic regression; the number of imputations conducted; the analysis model that is fitted to obtain estimates within each imputed dataset; and the method for combining estimates across imputed datasets [17]. If using an approach that incorporates an assumption about a difference in distribution between the missing and observed values (e.g., a NARFCS procedure), then, in addition to the above quantities, it is important to describe how the assumption is incorporated in the models used for the estimation procedure.

## Methods

The protocol for this scoping review has been published previously [7]. Briefly, we included observational studies that aimed to answer at least one causal research question using MI, published in *International Journal of Epidemiology, American Journal of Epidemiology, European Journal of Epidemiology, Journal of Clinical Epidemiology* and *Epidemiology* between January 2019 and December 2021. These journals were chosen as they are high ranking, general journals in epidemiology that we expected would capture current best practices in the use of MI for estimating ACEs from observational data. This selection of journals has been used previously in a systematic review of epidemiologic practice [24]. A full text search for the term “multiple imputation” was conducted on the journal websites, following the methodology of Hayati Rezvan et al [8]. Causal questions were identified if the study authors explicitly stated that they were estimating an ACE or if the study authors estimated an effect that was given, at least implicitly, a causal interpretation. Studies were excluded from the review if they met any of the following criteria: the study did not aim to answer a causal question, a clear research goal could not be identified, the primary purpose of the article was methodological development, the analysis was based on aggregated data, the article reported qualitative research, the study exposure was assigned to participants by investigators (i.e. a trial), or the study was retracted. The most recent search was performed on 10^th^ June 2022.

A random sample of 10 articles were independently screened and reviewed by two reviewers (RM and KL) to develop the data collection instrument. One reviewer (RM) screened and reviewed all articles. Double data extraction was independently completed for 10% of articles (RM and KL). In addition, a second reviewer (CN or KL) screened articles when there was uncertainty about the inclusion criteria and reviewed articles when there was uncertainty about the information being extracted. Disagreements between reviewers were resolved via discussion with a third reviewer.

A summary of the data extraction items and a copy of the data extraction questionnaire are provided in Table 1 and the Supplementary Material, respectively, of Mainzer et al [7]. Briefly, for each study included in the review, data were extracted on the following: study characteristics; the quantity of missing data; the missing data assumptions made and whether these assumptions were justified; details of the primary analysis and whether or not the primary analysis was justified based on missing data assumptions; details of any secondary/sensitivity analysis conducted that handled the missing data differently from the primary analysis and its justification; and details of the MI implementation. For each study, we defined the “inception sample” as the set of participants who met eligibility criteria for inclusion in the study to answer the research question of interest, where eligibility criteria do not include any requirement for variables to be complete, and the “analysis sample” as the participants who were included in the analysis to answer the research question of interest. Defining both the inception and analysis sample was necessary as we cannot always establish the size of the inception sample (either because authors neglect to report it, or because they were unable to define it in cases where eligibility data are themselves missing, e.g. for a study using electronic medical records in which patients may not appear in the database unless eligibility-defining measurements have been taken), and therefore needed a way to specify denominators for calculating percentages of missing data (which was the number in the analysis sample). Since our review only included studies with missing data within the analysis sample, the subset with complete cases was always smaller than the analysis sample. Extracted items were summarised using descriptive statistics. Data cleaning and analysis was performed in R [25]. Reporting follows the Preferred Reporting Items for Systematic Reviews and Meta-Analyses extension for Scoping Reviews checklist [26].

## Results

### Screening process

**Fig. 1:**
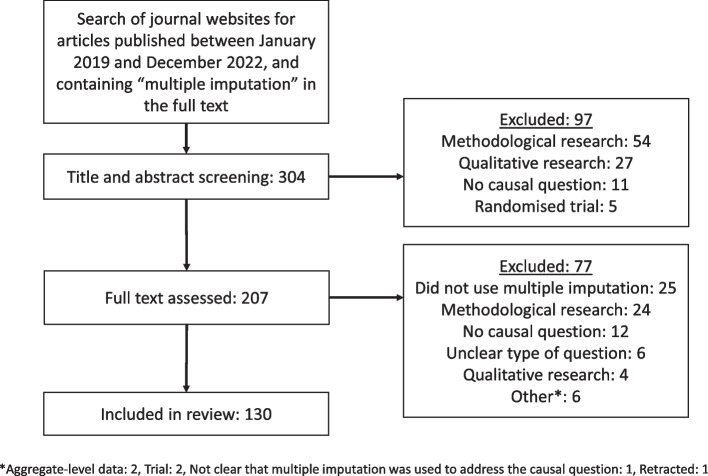
Article screening process

Figure 1 presents a flow diagram of the article screening process. Of the 304 papers that met the inclusion criteria, 130 papers were included in this review [27–156]. There were 14 articles that were screened by a second reviewer due to uncertainty about inclusion criteria. Double data extraction was completed for a further 14 articles. All disagreements were resolved via discussion. Minor changes were made to the review protocol to accommodate unanticipated challenges in data extraction (described in Additional file 1).

### Study characteristics

Study characteristics are summarised in Table 1. Most papers included in this review were published in *American Journal of Epidemiology* (38%) or *International Journal of Epidemiology* (26%). The most common study design was a prospective longitudinal study (65%), followed by a retrospective analysis of routinely collected data (12%). The most common outcomes used for analyses were binary (35%) and time to event (38%). Few studies made their causal aim explicit (25%) or presented a DAG to depict causal assumptions (31%). However, most studies identified a set of variables to control for confounding (82%) and almost all studies estimated an effect using a regression model (or a more sophisticated causal effect estimation method such as g-computation) with adjustment for a set of covariates, implicitly or explicitly assumed to be confounders (99%).

**Table 1:** Summary of study characteristics for the 130 included papers

**Characteristic**	**n (%)**
Publication year	
2019	47 (36%)
2020	45 (35%)
2021	38 (29%)
Journal^a^	
American Journal of Epidemiology	50 (38%)
Epidemiology	24 (18%)
European Journal of Epidemiology	21 (16%)
International Journal of Epidemiology	34 (26%)
Journal of Clinical Epidemiology	1 (1%)
Study design	
Prospective longitudinal study	85 (65%)
Retrospective analysis of routinely collected data	15 (12%)
Pooled cohort analysis	9 (7%)
Case-control study	7 (5%)
Cross-sectional study	5 (4%)
Case-cohort study	2 (2%)
Other^b^	7 (5%)
Type of outcome used for analysis	
Binary	45 (35%)
Categorical (excluding binary)	3 (2%)
Continuous	33 (25%)
Time to event	49 (38%)
Causal question inclusion criteria^c^	
Explicitly stated interest in a causal effect	33 (25%)
Estimate was given a causal interpretation	130 (100%)
Typical signals of a causal analysis^c^	
A directed acyclic graph was used to depict causal assumptions	40 (31%)
A set of variables were identified to control for confounding	106 (82%)
Effect was estimated using a regression model with adjustment for a set of covariates^d^	129 (99%)

^a^Number of papers published between January 2019 and December 2021 based using a Pub Med search for (("2019/01/01"[Date - Publication] : "2021/12/31"[Date - Publication])) AND ("*Journal name*"[Journal]): American Journal of Epidemiology, 876; Epidemiology, 496; European Journal of Epidemiology, 370; International Journal of Epidemiology, 814; Journal of Clinical Epidemiology, 996

^b^Secondary analysis of trial data (*n* = 2); prospective follow-up of cohort recruited for trial (*n* = 2); pooled analysis of data from case-control and cohort studies (*n* = 1); pooled analysis of data from case-control studies (*n* = 1); transportability study using data from 4 clinical trials and 1 observational cohort (*n* = 1)

^c^Categories are not mutually exclusive

^d^One study used structural equation modelling seemingly without adjustment for covariates, although a causal conclusion was made

### Missing data summaries

**Fig. 2:**
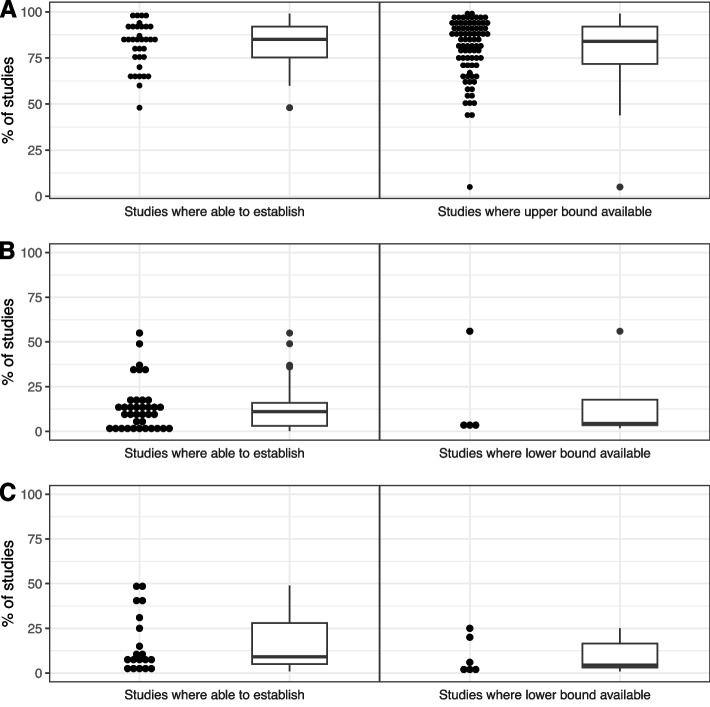
Dot plots and histograms showing the extent of missing data as a proportion of all participants in the analysis sample (see text for definition). **A**) no missing data (complete cases); **B**) missing values in the exposure; **C**) missing values in the outcome. Left panels: restricted to studies where the percentage could be established; right panels: restricted to studies where the exact percentage could not be established but a conservative bound on the percentage could be established

The reported quantity of missing data is summarised in Table 2 and Fig. 2. The size of the inception sample could not be established in 38% of studies, and 83% of studies derived an analysis sample by excluding individuals with missing data in specific variables. The percentage of complete cases could be established in just 34/130 (26%) studies (median, 25^th^ – 75^th^ percentiles: 85%, 75% – 92%), although an upper bound on the percentage of complete cases that was tighter than 100% (indicating the maximum possible percentage of complete cases given the missing data summaries provided) could be established for another 80/130 (62%) studies (median upper bound, 25^th^-75^th^ percentiles: 84%, 72% – 92%). Almost all studies (88%) incurred missing data in multiple variables in the analysis sample (despite most studies already arriving at an analysis sample by excluding individuals with missing data in specific variables).

**Table 2:** Amount of missing data. Summaries are n (%) unless stated otherwise

**Characteristic**	**Summary**
Able to establish the size of the inception sample^a^	
Yes	81 (62%)
No^b^	49 (38%)
Analysis sample was defined by excluding individuals with missing data in specific variables	
Yes	108 (83%)
No	22 (17%)
Complete cases	
Able to establish the % of complete cases % of complete cases, median (25^th^–75^th^ percentiles)	34 (26%) 85% (75% – 92%)
Only able to establish an upper bound on the % of complete cases Upper bound on % of complete cases, median (25^th^–75^th^ percentiles)	80 (62%) 84% (72% – 92%)
Not able to establish the percentage of complete cases	16 (12%)
Missing values in the exposure	
Yes, and able to establish the % of missing values % of missing values, median (25^th –^ 75^th^ percentiles)	39 (30%) 11% (3% – 16%)
Yes, but only able to establish a lower bound on the % of missing values Lower bound on % of missing values, median (25^th –^ 75^th^ percentiles)	4 (3%) 5% (4% - 18%)
Yes, but unable to establish the % or a lower bound on the %	7 (5%)
No	70 (54%)
Unclear	10 (8%)
Missing values in the outcome^c^	
Yes, and able to establish the % of missing values % of missing values, median (25^th –^ 75^th^ percentiles)	19 (15%) 9% (5% – 28%)
Yes, but only able to establish a lower bound on the % of missing values Lower bound on % of missing values, median (25^th –^ 75^th^ percentiles)	6 (5%) 5% (3% – 17%)
Yes, but unable to establish the % or a lower bound on the %	6 (5%)
No	91 (70%)
Unclear	8 (6%)
Missing values in the covariates	
Yes, in 2 or more	109 (84%)
Yes, in 1 covariate only	7 (5%)
No	7 (5%)
Unable to establish	7 (5%)
Multivariable missingness within analysis sample	
Yes	114 (88%)
No	8 (6%)
Unable to establish	8 (6%)

^a^Inception sample defined as the participants who met eligibility criteria for inclusion in the study to answer the research question of interest, where eligibility criteria do not include any requirement for variables to be complete

^b^Includes 5 studies where analyses were conducted separately by sub-groups (e.g., sex), but the inception sample for the sub-group could not be identified even though the inception sample for the entire group may have been provided

^c^Time-to-event outcomes were not considered to be missing data (we did not treat censored data as missing) except for in two studies where authors explicitly stated that the outcome was imputed

### Missing data assumptions

Missing data assumptions are described in Table 3. Most studies (66%) omitted a statement about missing data assumptions entirely. Of the 44 studies that did provide an explicit or indirect statement about missing data assumptions, 35/44 (80%) stated the MAR assumption, 2/44 (5%) stated the MCAR assumption and 6/44 (14%) alluded to data being “not MCAR” but did not distinguish between MAR and MNAR. Eleven of the 44 (25%) studies that provided a statement about missing data assumptions provided a justification for their missing data assumptions (described in Table 3, footnote 3). For example, justifications that were provided for the 6 studies that assumed data were MAR included: describing characteristics associated with missingness and/or conducting formal hypothesis tests (*n* = 4); examining the missingness pattern (*n* = 1), and; because study participants moved homes and/or were impossible to locate (*n* = 1). Of the 130 studies in the review, 31 (24%) linked the justification for the primary analysis to the missing data assumptions.

**Table 3:** Assumptions about the missing data mechanism

**Characteristic**	**Summary**
Missing data assumptions^a^	
No statement of missing data assumptions was provided	86 (66%)
Data were assumed to be MAR	35 (27%)
Data were assumed to be not MCAR	6 (5%)
Data were assumed to be MCAR	2 (2%)
A comprehensive description of missing data assumptions was provided, e.g., using an m-DAG	0 (0%)
Other^b^	1 (1%)
Justification provided for missing data assumptions (as % of papers that made a statement about missing data assumptions, *n* = 44)	
Yes^c^	11 (25%)
No	33 (75%)
Justified the primary analysis using missing data assumptions	
Yes	31 (24%)
No	98 (75%)
Other^d^	1 (1%)

*Abbreviations*: *MAR *missing at random, *MCAR *missing completely at random, *MNAR *missing not at random, *m-DAG *missingness directed acyclic graph

^a^The assumption may have been stated explicitly or made indirectly. For example, explicit statements of the MAR assumption include: “We assumed the missing at random assumption held and is reasonable”, [112] and “We imputed data using multiple imputation by chained equations under the assumption that data were missing at random” [140]. Indirect statements of the MAR assumption include “This multiple imputation approach assumes missing at random”, [93] and “We first imputed missing values using multiple imputation by chained equations, which assumes the data are missing at random conditional on the variables in the imputation model” [120]

^b^Data assumed to be “MCAR, conditional on age and ethnicity” (*n* = 1)

^c^Two studies justified assuming that data were MCAR; justifications included adding the questionnaire to the study after the study began (*n* = 1) and a lack in data registration (*n* = 1). Three studies justified assuming that data were not MCAR; justifications included clinicians ordering tests according to glucose level (*n* = 1), and describing characteristics associated with missingness (*n* = 2). Six studies justified assuming that data were MAR; justifications included describing characteristics associated with missingness and/or conducting formal hypothesis tests (*n* = 4), examining the missingness pattern (*n* = 1) and because children moved homes and/or were impossible to locate (*n* = 1)

^d^Justified MI to improve efficiency in the estimators

### Primary and sensitivity analyses

Details of the primary and secondary/sensitivity analyses are described in Table 4. Most studies (79%) used MI as the primary analysis method and approximately half (69/130, 53%) of the studies conducted a secondary analysis that handled the missing data differently. Of the 69 studies that conducted a secondary analysis, 70% of studies either provided no justification for conducting the secondary analysis or justified the secondary analysis as a sensitivity analysis without describing to what aspect of their primary analysis they were assessing sensitivity. A further 17/69 (25%) studies provided a vague justification for the secondary analysis, including to examine the influence of missing data (6%), to examine the impact of the missing data method (10%), and to address possible selection bias (9%). 88% of studies that conducted a secondary analysis performed both a CCA and an MI analysis; of these, only 3 studies (5%) observed a substantial difference between CCA and MI estimates. One study (1%) conducted an “extreme case” analysis that involved single imputation of the outcome under two extreme scenarios, thereby incorporating an external assumption about a difference in distribution between the missing and observed outcome data. However, no studies used a model-based approach such as a NARFCS procedure or elicited external information from subject-matter experts about the difference in distribution between the missing and observed data.

**Table 4:** Primary and secondary analyses

**Characteristic**	**n (%)**
Method used for the primary analysis	
Standard MI	80 (62%)
Standard MI, combined with weighting^a^	23 (18%)
CCA	21 (16%)
Other^b^	6 (5%)
Secondary analysis conducted that handled the missing data differently	
Yes	69 (53%)
No	61 (47%)
Method used for the secondary analysis (as % of papers that conducted a secondary analysis, *n*=69)	
Standard MI	27 (39%)
Standard MI, combined with weighting^a^	2 (3%)
CCA	26 (38%)
CCA, combined with weighting^a^	6 (9%)
Conducted more than two secondary analyses^c^	8 (12%)
Justification for the secondary analysis, (as % of papers that conducted a secondary analysis, *n*=69)	
Not provided	25 (36%)
As a sensitivity analysis (without further justification)	23 (33%)
To examine the influence of missing data	4 (6%)
To examine the impact of the missing data method	7 (10%)
To address possible selection bias	6 (9%)
To examine robustness to parametric modelling assumptions	1 (1%)
To examine robustness to causal assumptions about the missing data mechanism	0 (0%)
Other^d^	3 (4%)
Conducted both a CCA and an MI analysis, regardless of whether weighting was used or not (as % of papers that conducted a secondary analysis, *n*=69)	
Yes	61 (88%)
No^e^	8 (12%)
Observed a substantial difference between MI and CCA estimates, (as % of papers that conducted both a CCA and a MI analysis, *n*=61)	
Yes	3 (5%)
No	58 (95%)

^a^Weights were used to address selection bias due to loss to follow up or censoring. Excludes weights that were used to address confounding bias

^b^Treated “missing” as an additional category, with or without weighting (*n* = 3); Single median imputation to obtain exposure (*n* = 1); Single mean imputation for variables with >25% missing values (*n* = 1); MI for one covariate with >25% missing values and single (median/mode) imputation for variables with less than 5% missing values (*n* = 1)

^c^Described in Additional file 1, Supplementary Table 1

^d^As a sensitivity analysis to examine the robustness of findings to statistical assumptions without stating which statistical assumptions (*n* =1); as a sensitivity analysis to address possible selection bias and to exploit information in incomplete record participants (*n* = 1); Estimates were presented from both MI and a CCA after fitting two different models (one weighted and one unweighted). No justification was provided for conducting both MI and CCA analyses, but fitting two models was justified by seeing whether the choice of model impacted results and fitting models with and without weights was conducted to see how weighting affected the results (*n* = 1)

^e^Standard MI, with and without weighting (*n* = 4); Standard MI, with weighting, with and without imputation of exposure (*n* = 1); Standard MI, with and without inclusion of the outcome in the imputation model (*n* =1); Three versions of single imputation and standard MI (*n* = 1); Missing treated as an additional category, last value carried forward and standard MI (*n* = 1)

### MI implementation

The details of the MI implementation are described in Table 5. Most studies (71%) reported the number of imputations (median, 25^th^-75^th^ percentiles: 20, 3 – 100). Multivariate imputation by chained equations was the most used imputation method (67% of studies), but the imputation method was unclear for a further 25% of studies. MI was most often conducted in Stata or R. In more than half of the studies it was unclear whether all analysis variables were included in the imputation procedure (58%), whether auxiliary variables were used in the imputation procedure (55%), and whether interactions were included in the imputation model (57%). Of the 87 studies that reported using multivariate imputation by chained equations, 18 (21%) reported the type of models that were used in the imputation procedure. In approximately two-thirds (65%) of studies, the method that was used to obtain a final MI estimate and its standard error was not stated and could not be deduced from the description in the paper. We assume that most studies would use Rubin’s rules to produce a final estimate and standard error, although alternative approaches are available (see, e.g., [157]).

**Table 5:** Multiple imputation implementation. Summaries are n (%) unless stated otherwise

**Characteristic**	**Summary**
Reported number of imputations	
Yes	92 (71%)
No	38 (29%)
Number of imputations, median (25th - 75th percentiles)	20 (3 – 100)
Multiple imputation method	
Multivariate imputation by chained equations	87 (67%)
Multivariate normal imputation	6 (5%)
Other^a^	4 (3%)
Unclear	33 (25%)
Software package used for conducting the multiple imputation analysis	
Stata	40 (31%)
R	33 (25%)
SAS	26 (20%)
SPSS	1 (1%)
Other^b^	14 (11%)
Unclear	16 (12%)
All analysis variables included in imputation model	
Yes	35 (27%)
No	20 (15%)
Unclear	75 (58%)
Auxiliary variables included in imputation model	
Yes	42 (32%)
No	16 (12%)
Unclear	72 (55%)
Interactions included in imputation model	
Yes	2 (2%)
No	54 (42%)
Unclear	74 (57%)
Reported type of models used for imputation (as % of papers that used multivariate imputation by chained equations, *n*=87)	
Yes	18 (21%)
No	69 (79%)
Stated how a final estimate and standard error were obtained	
Either stated, provided code or method could be deduced from software description	45 (35%)
Not stated	85 (65%)

^a^Imputation performed using a bootstrapping-based algorithm for panel data in R package Amelia II (*n* = 1), imputation performed in the pan package mitml for multilevel data (*n* = 1), referenced a paper where the MI methods are described rather than providing a description (*n* = 1), used a multiple imputation analysis for exposure and covariates without stating what the analysis was, and used Kaplan-Meier multiple imputation for the outcome as part of a sensitivity analysis (*n* = 1)

^b^Study used two software packages for analysis but it was not clear which package was used for MI (*n* = 13), NORM software (*n* = 1)

## Discussion

We systematically reviewed the literature to assess the current state of practice in using MI for estimation of causal effects from incompletely observed observational data. We focussed on four key areas: missing data summaries, missing data assumptions, primary and sensitivity analyses, and MI implementation. Overall, we found that most studies are not reporting missing data, and missing-data-related assumptions, decisions, or analyses with sufficient clarity.

Similarly to other reviews [11–13], we found that the analysis sample is often arrived at by excluding individuals with missing data in certain variables, for example, by using eligibility criteria that require key variables to be completely observed. This is worrying as the preliminary exclusion of individuals may lead to selection bias [158]. It also means that the full extent of missing data is difficult to quantify due to difficulty in identifying the inception sample. Therefore, for the purposes of reporting the amount of missing data in this review, we considered the amount of missing data within the analysis sample only. However, identifying the exact amount of missing data within the well-defined analysis sample was also often difficult because summaries were frequently reported per variable without describing missing data patterns.

Details of the assumptions made about the missing data mechanism were often lacking and, when provided, not justified appropriately. A statement of assumptions about the missingness mechanism was provided for just one-third (33%) of studies. This is a marginal improvement over what was found in the reviews conducted by Mackinnon (2010), where 8/50 (16%) observational studies provided a statement that data were MAR, [9] and Rezvan et al. (2015), where 7/30 (23%) observational studies stated or described the assumed missing data mechanism, [8] and illustrates that concerted effort is still needed to improve transparency around missing data assumptions. When a statement about the missing data mechanism was provided, most studies said they assumed data were MAR, but justifications for missingness assumptions were provided in just 11 studies. When they were provided, justifications were generally vague or incomplete. As highlighted in the Introduction, the MCAR/MAR/MNAR assumptions are difficult to interpret and assess in the context of multivariable missingness, so it is not surprising that we found lacking or incomplete justifications for these assumptions. Of note, no study provided a comprehensive description of missing data assumptions, for example, using an m-DAG. Furthermore, the omission of a statement of missing data assumptions entirely from most studies suggests that the critical link between missing data assumptions and estimation methods is not generally appreciated. When missing data assumptions were used to guide the choice of MI as the primary analysis, the most common justification for using MI was because data were assumed to be MAR (without justifying the MAR assumption).

Most studies in this review used standard MI for the primary analysis. Approximately half of the studies conducted a secondary analysis that treated the missing data differently from the primary analysis, but the reason for doing so was almost always omitted or unclear. When studies did carry out two analyses that handled the missing data differently, it was common to conduct both a CCA and MI. Without justification, it is not clear why such an analysis is warranted. It may be to examine the sensitivity of ACE estimates to causal assumptions made about the missing data mechanism for the primary analysis. We speculate another motivation for such an analysis may be the misconception that a CCA is the “normal” approach to dealing with missing data while standard MI provides a more sophisticated analysis that allows you to assess whether the missing data were really an issue or not. However, if under plausible missingness assumptions neither standard MI nor CCA can provide unbiased estimation, then it would be incorrect to conclude that the missing data “had little impact” on the results. In other words, when there is no unbiased estimate to compare against, the impact of the missing data remains unknown. Of the 61 studies that conducted both a CCA and MI analysis, only 3 (5%) studies observed a substantial difference between MI and CCA estimates. Just one study conducted an analysis that incorporated assumptions about a difference between the missing and observed data distributions. Despite being an area of recent methodological development, our finding that such analyses are not being performed often is similar to findings from previous reviews, see e.g. [8, 159].

MI is increasingly recognised as a method for estimation that needs to be tailored to the target analysis, for example, by careful selection of which variables to include in the imputation model and in what form [6], and by examining the implications of assumptions encoded in an assumed m-DAG [3]. However, the findings from the current review suggest that there is room for improvement in the reporting of MI implementation. For example, certain aspects of the imputation model form were reported just over half of the time despite being needed to judge the appropriateness of the MI model and ensure the analysis can be reproduced.

As noted earlier, several useful frameworks and guidelines around the handling and reporting of missing data are available in the literature. However, the findings from this review suggest that the available guidelines are not being followed. Journals could play more of a role in ensuring appropriate handling and reporting of missing data. Furthermore, although there is growing guidance around using MI in causal inference (see e.g. Chapter 13 of [160]), further guidance is needed when the research question, assumed m-DAG, and/or analysis model are more complex than those considered in the guidance documents, for example, in the context of causal mediation analysis.

The strengths of this review are that it documents the current practices in the use of MI for estimating ACEs from incomplete observational data. Our review followed a clear, pre-specified protocol [7], and by including articles in top general epidemiology journals, we tried to capture current best practice. Furthermore, the analysis conducted for the current study is entirely reproducible as all data and code are available on GitHub: github.com/rheanna-mainzer/MI-scoping-review. This review has several limitations. Authors may have chosen not to provide details on all aspects of handling missing data that we examined, for example, due to strict journal word limits. However, all accompanying supplementary material was also reviewed and used for data extraction. Most of the data extraction was performed by a single reviewer (RM), with double data extraction performed for 10% of studies, so there may be some extraction errors. Also, it may have been useful to extract additional items or extract items in more detail to better capture the variety of analyses undertaken. However, additional notes on each paper were recorded and are available as part of the complete dataset on GitHub. Lastly, by limiting to five top general epidemiology journals, our results may not reflect papers published in other journals.

## Conclusion

The message from our review is clear: there is a need for greater clarity in the conduct and reporting of causal effect estimation using MI with incomplete observational data. Researchers are encouraged to follow the guidance that is available regarding the handling of missing data, to move beyond the MCAR/MAR/MNAR framework and adopt a more transparent approach for outlining missing data assumptions, to use missing data assumptions to justify the estimation method, and to report their assumptions, methods and results systematically.

## Supplementary Information

Supplementary Material 1

## Data Availability

The datasets supporting the conclusions of this article are available from RM’s GitHub repository: github.com/rheanna-mainzer/MI-scoping-review.

## Abbreviations

ACE: Average causal effect
CCA: Complete case analysis
DAG: Directed acyclic graph
MAR: Missing at random
MCAR: Missing completely at random
m-DAG: Missingness directed acyclic graph
MI: Multiple imputation
MNAR: Missing not at random
NARFCS: Not-at-random fully conditional specification
