# Supplementary material for: Gaps in the usage and reporting of multiple imputation for incomplete data: findings from a scoping review of observational studies addressing causal questions
Source: BMC Med Res Methodol. 2024 Sep 4;24:193. doi: 10.1186/s12874-024-02302-6 (PMC11373423; doi:10.1186/s12874-024-02302-6)
Supplement: Supplementary file 1 — Supplementary Material 1 [file 12874_2024_2302_MOESM1_ESM.docx]

**Gaps in the usage and reporting of multiple imputation for incomplete data: Findings from a scoping review of observational studies addressing causal questions**

**Additional file 1**

Rheanna M. Mainzer*^1,2^, Margarita Moreno-Betancur^1,2^, Cattram D. Nguyen^1,2^, Julie A. Simpson^3,4^, John B. Carlin^1,3^, Katherine J. Lee^1,2^

^1^Clinical Epidemiology and Biostatistics Unit, Murdoch Children’s Research Institute, Parkville, Victoria 3052, Australia

^2^Department of Paediatrics, The University of Melbourne, Parkville, Victoria 3052, Australia

^3^Centre for Epidemiology and Biostatistics, Melbourne School of Population and Global Health, The University of Melbourne, Parkville, Victoria 3052, Australia

^4^Nuffield Department of Medicine, University of Oxford, Oxford, UK

*Corresponding author: [rheanna.mainzer@unimelb.edu.au](mailto:rheanna.mainzer@unimelb.edu.au)

**Contents:**

- Changes to review protocol
- Supplementary Table 1: Description of the primary and secondary analyses when studies performed more than one secondary analysis.

**Changes to review protocol**

1. Exclusion criteria were expanded to include retracted articles.
2. The following additional items were extracted during a systematic check of the extracted data:
   1. What type of outcome was used for analysis? Multiple choice selection from the following options: binomial, categorical (not binary), continuous, or time-to-event.
   2. If multivariate imputation by chained equations was used, what type of models were used? Open-ended response.
   3. How were estimates combined across imputed datasets? Open ended response.
3. The item “Did authors address the potential for data to be MNAR?” was difficult to measure as selection bias was commonly described as a study limitation in vague terms. Rather than summarise this item in the tables, information on the studies that conducted an analysis incorporating assumptions about a difference in distribution between the missing and observed data have been summarised in text.
4. Study funding was not extracted as funding source was deemed not applicable for this review of methodology.

**Supplementary Table 1.** Description of the primary and secondary analyses when studies performed more than one secondary analysis.

| **Primary analysis** | **Secondary analyses** |
| --- | --- |
| Standard MI | Standard MI combined with weighting using two approaches for calculation of weights |
| Standard MI (covariates imputed) | CCA, standard MI (covariates and exposure imputed) |
| Standard MI combined with weighting | Standard MI, CCA, CCA combined with weighting |
| Single mean imputation for variables with >25% missing data | Standard MI, single minimum value imputation, single maximum value imputation. |
| Standard MI combined with weighting | Standard MI, CCA, CCA combined with weighting |
| Standard MI | Standard MI but excluding one study with >25% missing data for exposure (data collected from multiple studies), CCA |
| CCA | Standard MI where the time-to-event outcome was imputed using a Kaplan-Meier-based imputation method, single imputation of outcome under two extreme scenarios |
| Treated “missing” as an additional category | Single imputation using last value carried forward, standard MI |
